# Domestic violence related disclosure among women and girls in Ethiopia: a systematic review and meta-analysis

**DOI:** 10.1186/s12978-019-0845-z

**Published:** 2019-12-23

**Authors:** Berhanu Boru Bifftu, Berihun Assefa Dachew, Bewket Tadesse Tiruneh, Lemma Derseh Gezie, Yonas Deressa Guracho

**Affiliations:** 1 University of Gondar College of Medicine and Health Science, School of Nursing, Gondar, Ethiopia; 2 Department of Epidemiology and Biostatistics, Institute of Public Health, College of Medicine and Health Sciences, University of Gondar, Gondar, Ethiopia; 3 The University of Queensland, Institute for Social Science Research, Indooroopilly Qld, 4068 Australia; 4 College of Medicine and Health Science, Department of psychiatry Ethiopia, Bahir Dar University, Bahir Dar, Ethiopia

**Keywords:** Barriers, Disclosure, Domestic violence, Ethiopia, Girls, Women

## Abstract

**Background:**

Domestic violence is common public health problem. Domestic violence related disclosure is an important first step in the process of prevention, control and treatments of domestic violence related adverse effect. Thus, this systematic review and meta-analysis aimed to determine the pooled prevalence of domestic violence related disclosure and synthesize its associated factors.

**Methods:**

We followed the PRISMA Guidelines to report the results of the finding. Databases including PubMed, Cochrane Library and Web of Sciences were searched. The heterogeneity between studies was measured by the index of heterogeneity (I^2^ statistics) test. Funnel plots and Egger’s test were used to determine publication bias. Moreover, sensitivity analysis was carried out. To calculate the pooled prevalence, a random effects model was utilized.

**Results:**

Twenty one eligible studies were included in this systematic review and meta-analysis. The pooled prevalence of domestic violence related non-disclosure was found to be 36.2% (95% CI, 31.8**–**40.5%). Considering violence as normal or not serious, shame, embarrassment and fear of disclosure related consequences were the common barriers for non-disclosure.

**Conclusion:**

More than one third of women and girls were not disclosed their experience of domestic violence. The finding of this study suggests the need of evaluation and strengthening of the collaborative work among different sectors such as: policy-makers, service providers, administrative personnel and community leaders including the engagement of men partner. This study also suggests the needs of women empowerments against the traditional belief, attitude, and practice.

## Plain English summary

Domestic violence is common public health problem. In the prevention, control and treatment of domestic violence related adverse effect; disclosure is an important first step particularly for those who experienced. In Ethiopia, the available individual study findings regarding domestic violence related disclosure and its associated factors were inconsistent. Thus, this systematic review and meta-analysis aimed: to synthesize barriers to domestic violence non-disclosure and to determine the pooled prevalence of non-disclosure. To synthesis the evidence, databases such as PubMed, Cochrane Library and Web of Sciences were searched. Twenty one eligible studies were included for the analysis. The results of this study showed that more than one third of survivors of domestic violence were not disclosed their experience of violence. Barrier like perceptions of domestic violence as normal or not serious, shame, embarrassment and fear of disclosure related consequences were the identified common barriers for the non-disclosure of the violence. The finding of this study suggests the need of evaluation and strengthening of the collaborative work among different sectors such as: policy-makers, service providers, administrative personnel and community leaders including the engagement of men partner. This study also suggests the needs of women empowerments against the traditional belief, attitude, and practice.

## Background

Violence against Women and Girls (VAWGs) is one of public problem, affects the individual, family and community life regardless of their age, race, nationality and socio-economic status [1, 2]. Apart from the violations of human rights, domestic violence (DV) is associated with various poor health outcomes for the new born such as: low birth weight, premature birth, placental damage, fetal trauma, preterm labor; and among the women and girls include: suicide, homicide, mental illness, physical injuries, disability Moreover, DV also associated with various poor reproductive health condition such as: unintended pregnancy, induced abortion, bleeding, HIV and other sexual transmitted infections [1–4]. Globally, nearly one in every three women experiences DV at some point in their life [5]. Of this, the highest prevalence was found in African (37%) [5]. In Ethiopia, domestic VAW is common women’s life experience with an estimated prevalence ranged from 50 to76.5% during lifetime and 30 to72.5% for the past 12 months [6–11]. For appropriate and effective policy responses to prevent and address the adverse effects of DV an accurate and complete understanding the gap between the magnitude of DV and its disclosure is mandatory, yet many women who exposed violence were not disclose/seek help. For example, according to the World Health Organization (WHO) multi-country study, 55–95% of women who had experienced physical or sexual IPV have never sought help from formal institutions [1, 2, 12]. Factors such as gender norms, poverty, denied access to education, lack of autonomy, inequitable gender attitudes, women’s acceptance wife beating and partner alcohol use [2, 3, 13, 14], socio-cultural normal [1, 15], shame, embarrassment [16, 17], fear of disclosure related consequences [15, 18] and economic dependence were associated with non-disclosure [15, 16]. This is not different for Ethiopia, where the prevalence of DV ranged from 30 to72.5%, of this up to 93% of them were not disclosed to anyone [1, 2, 9–11, 19–24] and from those who disclosed their experiences to anybody, only 10% of them were to the formal services like police and health care professionals [1, 3, 25–29]. According to the 2016 Ethiopian Demographic and Health Survey (EDHS), of those women who experienced physical, or sexual or both violence 66% of them were not told to any one [14]. In response to this, the government of Ethiopia has been incorporated the issue of women’s right and gender equality in the family law, [13, 30] criminal law and constitution [13, 31]. For the management of DV-related non- disclosure and its adverse effects, epidemiological determination of its magnitude and contextual identifications of barriers/associated factors is important. To date, numbers of studies have investigated the prevalence of DV-related disclosure. However, a great variability was found in the reported results [1, 2, 6, 15–18, 32–44]. Additionally, the findings related to DV-related disclosure have not been reviewed in a comprehensive manner. Hence, the present study is the first systematic review and meta-analysis of DV-related disclosure and its associated factors in Ethiopia. Thus, this systematic review and meta-analysis aimed (i) to determine the pooled prevalence of DV-related disclosure and (ii) to synthesize its associated factors in Ethiopia.

## Methods

This systematic review and meta-analysis followed the Preferred Reporting Items for Systematic Review and Meta-Analysis (PRISMA) [45].

### Search strategy

The search and document retrieval strategy were intended to capture range of published and un published literature using databases including: PubMed, Cochrane Library and Web of Sciences. A combination of Medical Subject Headings (MeSH) thesaurus, text words and combining with appropriate Boolean operators were used. A comprehensive search strategy tailored to each databases was developed. No time and language restricted during the search strategy. Moreover, the reference lists of all articles were searched. Furthermore, Google Scholar was searched for gray literature and published paper in un indexed journals. The full electronic search strategy for one of a data base (PubMed) was searched using the search term: ((gender based violence [MeSH Terms]) OR (gender based violence) OR (domestic violence [MeSH Terms]) OR (domestic violence) OR (intimate partner violence [MeSH Terms]) OR (intimate partner violence) OR (spouses violence[MeSH Terms]) OR (spouses violence) OR (physical abuse [MeSH Terms]) OR (physical abuse) OR (physical violence [MeSH Terms]) OR (physical violence) OR (emotions violence[MeSH Terms]) OR (emotions violence) OR (emotions abuse[MeSH Terms]) OR (emotions abuse) OR (psychological violence [MeSH Terms]) OR (psychological violence) OR (psychological abuse [MeSH Terms]) OR (psychological abuse) OR (sex violence [MeSH Terms]) OR (sex violence) OR (sex abuse[MeSH Terms]) OR (sex abuse) OR (harassment [MeSH Terms]) OR (harassment) OR (intimidation[MeSH Terms]) OR (intimidation) OR (sexual assault [MeSH Terms]) OR (sexual assault) OR (sexual coercion[MeSH Terms]) OR (sexual coercion) OR (rape [MeSH Terms]) OR rape)) AND ((disclosure [MeSH Terms]) OR (disclosure) OR (help seeking [MeSH Terms]) OR (help seeking) OR (service utilization[MeSH Terms]) OR (service utilization) OR (coping mechanism [MeSH Terms]) OR (coping mechanism) OR (defense mechanism [MeSH Terms]) OR (defense mechanism) OR (woman’s response [MeSH Terms]) OR (woman’s response) OR (resilience [MeSH Terms]) OR (resilience)) AND ((Barriers [MeSH Terms]) OR (Barriers) OR (reasons [MeSH Terms]) OR (reasons) OR (associated factors [MeSH Terms]) OR (associated factors) OR (determinants factors [MeSH Terms]) OR (determinants factors)) AND Ethiopia), until February, 18, 2019.

### Definition of concepts

In this study, the following operational definitions were used: (i) domestic violence was defined as any violence whether physical, psychological and sexual, or any combination of the three, regardless of the legal status of the relationship among women and girls of all age, (ii) physical violence was defined as one or more intentional acts of physical aggression such as: pushing, slapping, throwing, hair pulling, punching, hitting, kicking or burning, perpetrated with the potential to cause harm, injury or death, (iii) psychological/emotional violence was defined one or more acts, or threats of acts, including shouting, controlling, intimidating, humiliating and threatening the victim, [1, 2], (iv) sexual violence is defined as the use of force, coercion or psychological intimidation to force the woman to engage in a sex act against her will, whether or not it is completed [46], and (v) disclosure/help seeking was defined as any conversation or sharing of information and/or actually used help from at least one of either formal (e.g legal service provider, police, health care provider) and/or informal source (e.g., family members, friends, coworkers, local leader relatives, neighbors, local leaders/ leader’s wives club, Non-Governmental Organizations, religious healers and coded as ‘Yes’/‘No’ [1, 2, 47].

### Selection of studies

All articles retrieved through search strategy were imported to EndNote X7 (Thomson Reuters, New York, USA). After excluding the duplicated studies from EndNote Library, the title and abstracts of the remaining articles were assessed independently by two reviewers (YDG and BTT). Disagreements were resolved by discussion. in collaboration with the third author (BBB). Conference abstracts, letters to editors, review, and commentary articles were excluded.

### Eligibility criteria

#### Participants

This review targets all human participants irrespective of age, setting (institution and community) and population (general population, high school/university students). Studies in which participants drawn from pregnant women, refugee and not living in Ethiopia were excluded.

#### Outcome measures

This review included studies that investigated the prevalence of DV related disclosure and its barriers/associated factors.

#### Study design

Observational studies (cross-sectional and cohort/longitudinal) were included in this systematic review and meta-analysis. Studies that focused on case reports, conference and abstracts were excluded.

#### Data extraction

A standardized, pre-piloted form was used to extract the required information. The included study characteristics were: author’s name, year of publication, setting (e.g., high school/university students and general community), measurement tool, sample size, case/reported prevalence and associated factors/barriers.

#### Quality assessment

Two review authors’ independently assessed the quality of included studies using the adapted Newcastle-Ottawa quality assessment tool for cross-sectional studies [48]. The adapted Newcastle-Ottawa quality assessment tool has three main parts (selection, comparability and outcome). The first part (selection) has five stars and assesses the methodological quality of the study. The second part of the tool evaluates the comparability of the study. The third part of the tool assesses the quality of the original article’s outcome with respect to the statistical analysis. Individual paper was graded with score ranged from zero to ten stars. The overall quality of each study was determined using the sum score of each star of the three parts. If the overall score of a paper was ≥6 out of 10, it is categorized as good quality and if the score fulfilling 50% of quality assessment criteria, it is medium and for score ≤ 4, it is defined as poor quality.

#### Data synthesis

The extracted data were entered into a Microsoft Excel Data base and then imported into STATA 14, that we installed on line packages for meta-analysis. For the quantitative analysis, random-effects model with 95% confidence interval (95% CI) was used to calculate the pooled prevalence [45, 49]. Test for Heterogeneity between the studies was assessed with Cochran’s Q statistic and the *I*^2^ statistics. I^2^ values greater than 50% were considered as indicative of substantial heterogeneity [46]. Evidence of publication bias was assessed using Egger’s test [50] and the visual inspection of the symmetry in funnel plots [51]. Meta-analysis of barriers/risk factors was not possible because of the inconsistent and insufficient independent variables. Thus, results are summarized and presented using texts, figures and tables.

## Results

**Fig. 1:**
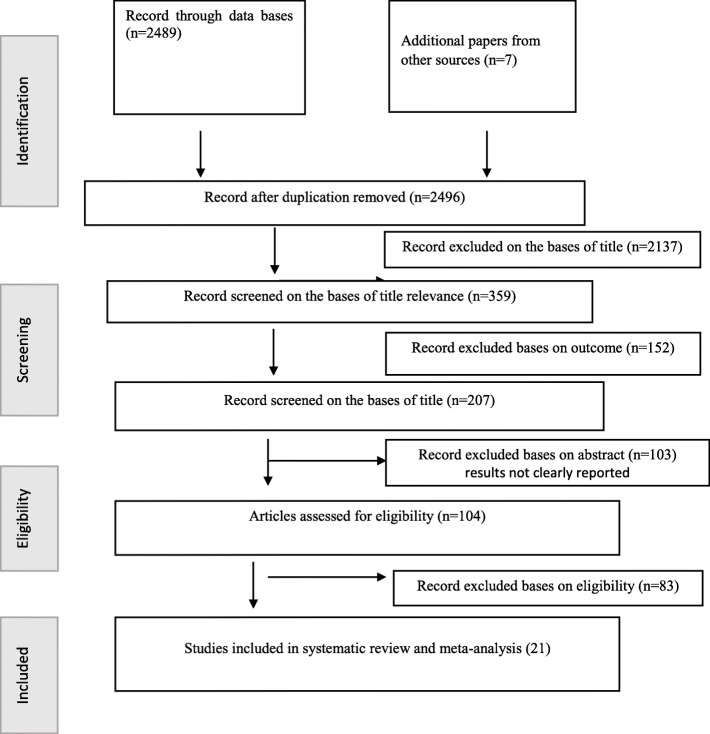
Flow diagram of the included studies

The literature search strategy resulted in 2496 recorded papers. Of this record, 2137 studies were excluded just by reading their titles. Of the remaining 359 studies, 152 were excluded on the bases of the outcome assessment. Moreover, 103 studies were excluded after reading the abstract because of unclearly reported outcome variables. Finally, 83 studies were excluded based on the eligibility criteria and the remaining 21 studies were included in the systematic review and meta-analysis (Fig. 1).

### Study characteristics

Of the 21 included studies, three were conducted among high school students, nine among university students and the remaining nine conducted among the general population of women and girls of age 15–49. These studies were conducted among four different regions: Southern Nation and Nationalities of People (*n* = 7), Amhara (*n* = 6), Oromia (*n* = 4), Addis Ababa (*n* = 2) and two national data. The sample sizes of included studies were ranged from 48 to 1478. Thirteen (62%) of the included studies used community based cross-sectional study design. Majority (57%) of the included outcome variable was sexual violence (*n* = 12).Physical violence was reported by five studies, one study reported the combination of sexual and physical violence and the remaining four studies reported overall violence. Majority (*n* = 11) of the studies utilized self-developed assessment tool to assess disclosure, its barriers/associated factors and preferred source of support. Eight studies used the WHO multi country assessment tool (Table 1).

**Table 1:** Characteristics of included studies (*n* = 21)

Author Year	Study setting	Design	Study population	Type of DV	Total no. of DV	No. of non-disclosure	Non-disclosure (%)
WHO, 2005 [1]	Overall country	Community based cross-sectional	General population	Physical	1478	576	39
Gossaye, 2003 [6]	SNNP	Community based cross-sectional	General population	Physical	1101	425	38.6
Shanko, 2013 [15]	SNNP	Community based cross-sectional	General population	All	166	133	80
H/mariam, 2008 [32]	Amhara	Community based cross-sectional	General population	Physical	256	170	66.4
Misganaw, 2013 [16]	Amhara	Community based cross-sectional	General population	Sexual	96	71	74
Bekele, 2015 [18]	Oromia	Institution based cross-sectional	University students	Sexual	66	44	66.7
Sendo, 2015 [17]	SNNP	Institution based cross-sectional	University students	Sexual	48	27	61
Mihrka, 2016 [33]	SNNP	Institution based cross-sectional	University students	Physical & sexual	86	31	36
Takele, 2014 [34]	Oromia	Institution based cross-sectional	University students	Sexual	101	17	17
Nimani, 2015 [35]	SNNP	Institution based cross-sectional	High school students	Sexual	109	13	12
Bekele, 2014 [36]	Oromia	Institution based cross-sectional	University students	Sexual	258	59	23
Worku, 2002 [37]	Amhara	Institution based cross-sectional	High school students	Sexual	141	124	89
Adinew, 2017 [52]	SNNP	Institution based cross-sectional	University students	Sexual	291	271	93
Assefa, 2010 [39]	SNNP	Institution based cross-sectional	High school students	Sexual	353	34	9.6
Abdurashid, 2013 [40]	AA	Institution based cross-sectional	General population	All	93	75	80.6
Tadesse, 2004 [41]	AA	Institution based cross-sectional	University students	Sexual	251	73	23
Shimekaw, 2013 [53]	Amhara	Institution based cross-sectional	University students	Sexual	200	38	19
Benti, 2015 [43]	Oromia	Institution based cross-sectional	University students	Sexual	179	61	34
Yigzaw, 2005 [29]	Amhara	Community based cross-sectional	General population	All	247	39	15.8
Semahegn, 2013 [42]	Amhara	Community based cross-sectional	General population	All	532	362	68
EDHS, 2016 [14]	Overall	Community based cross-sectional	General population	physical	1348	876	65

### Quality of included studies

The overall quality of included studies ranged from 4 to 8. Nineteen of the included studies had good quality and the remaining two studies had fair quality (Additional file 1).

### Prevalence of domestic violence related disclosure

**Fig. 2:**
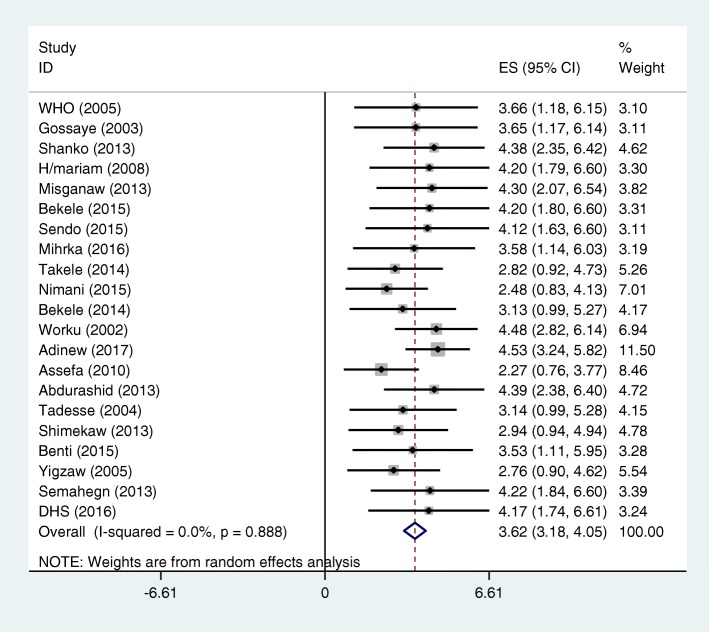
Forest plot assessing the prevalence of non-disclosure among women and girls survive of domestic violence in Ethiopia using random effect models with 95% CI

**Fig. 3:**
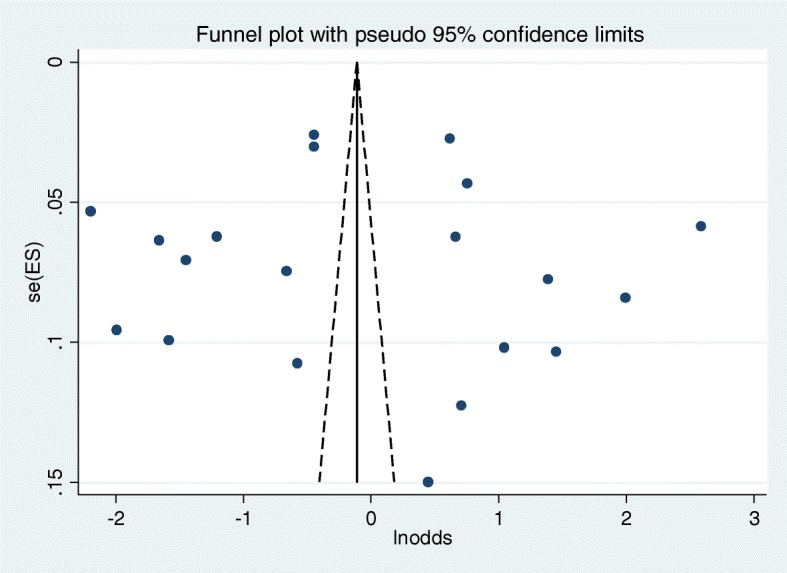
Funnel plot with pseudo 95% confidence interval that investigated the heterogeneity of the pooled prevalence of non-disclosure

In this systematic review and meta-analysis, the pooled prevalence of domestic violence related non-disclosure was found to be 36.2% (95% CI, 31.8**–**40.5%). No evidence of significant heterogeneity (I^2^ = 0% and *p* = 0.888) (Fig. 2) test and publication bias from the visual inspection of the funnel plot (Fig. 3) and the Egger’s test (*P* = 0.479). The result of sensitivity test also showed that none of the point estimates outside of the overall 95% confidence interval, confirming that there is no influential study. Thus, the pooled estimate of domestic violence related non-disclosure based on the 21 studies was important. Though evidence of heterogeneity does not support subgroup analysis, for the sake of clarity results are also presented by its subgroup of violence type, study setting and study population. Thus, result showed that higher pooled prevalence of DV-related non-disclosure among those women experiencing any physical violence (37.8% (95% CI: 25.5–50%) as compared to those women experiencing any sexual violence (34.9% (95% CI: 29.5–40.3%) and among those women experiencing both any physical/sexual violence (39% (95% CI: 29.7–48.6%%). The highest pooled prevalence of DV-related non-disclosure was also reported from general population (37.4% (95% CI: 30.5–44.5%%), and in Addis Ababa region (38% (95% CI: 23.4–52.7%%) (Table 2).

**Table 2:** Subgroup analyses by study: outcome, setting and population

Subgroup	Number of Studies	Pooled prevalence	95% CI	I^2^	*P*-value
Type of violence					
Physical	4	37.8%	25.5–50%	0.0%	0.538
Sexual	12	34.9%	29.5–40.3%	0.0%	0.984
Both	5	39.13%	29.7–48.6%	0.0%	0.733
Region/setting					
National	2	39.37%	20–56.7%	0.0%	0.763
SNNP	7	35.2	27–43%	20%	0.277
Amhara	6	37.8%	29.5–46%	0.0%	0.706
Oromia	4	33.3%	22.4–44%	0.0%	0.841
Addis Ababa	2	38%	23.4–52.7%	0.0%	0.404
Population					
General population	10	37.4%	30.5–44.5%	0.0%	0.967
University student	8	37.3%	30.4–44.2%	0.0%	0.685
High school student	3	31.7%	18.2–45.3%	0.0%	0.143

95% CI represents the 95% Confidence Interval for prevalence and I^2^ represents the prevalence of true heterogeneity

### Factors/barriers to disclosure

Out of the 21 included studies, 14 studies had information on factors/barrier/reasons for DV-related non-disclosure. In this systematic review and meta-analysis, several factors were identified as barriers to DV-related non-disclosure. The most common barriers were considering violence as normal/not serious for reporting [1, 15], shame and embarrassment [1, 16, 17, 38, 40], fear of consequences of DV-related disclosure [1, 15, 18, 32, 40], perceptions of reporting does not help [32, 43], lack of knowledge of of where to go [15] and what to do [15–17, 32, 34, 36, 38, 39]. In Ethiopia, women and girls are expected to tolerate any violence and keep/maintain her partners [26, 31, 54, 55] and if she shared the information to third party, it resulted in alienation, shame, embarrassment, or blame [20, 23]. Majority of the victims did not know where to go and did not consider disclosure of their experience has a solution, this in turn, women and girls survivors of violence not to report and seek help/justice against the perpetrators [30, 31]. The considerations of violence as normal or not serious, shame and fear associated with the consequences of DV-related disclosure make women and girls to be reluctant to seek help. This is also supported by belief that women are docile, submissive, patient, and tolerant of monotonous work and violence, for which culture is used as a justification [1, 26, 28] (Table 3).

**Table 3:** Factors/barrier for domestic violence non-disclosure

Author Year	Factors/barrier/reasons for domestic violence non-disclosure
WHO, 2005 [1]	Fear of consequence 53%, normal or not serious 37%
Gossaye, 2003 [6]	61(6%) fight back again to defend herself, 335 (30%) left home due to physical violence, 676 (61%) talk about the physical violence to someone
Shanko, 2013 [15]	Fear of exposing the issue 114 (68.7), fear of additional violence 90 (54.2), didn’t know where to go 38 (22.9), fear of divorce 36 (21.7), cultural tradition to accept it 30 (18.1) and other reason 96 (57.8)
H/mariam, 2008 [32]	Reporting does not help 20%, do not know how to report 15%, other 11%, fear of future anticipated violence 54%.
Misganaw, 2013 [16]	Embarrassment by 19 (29.7%), fear of rejection by legal bodies, lack of awareness where to report, fear of retribution and concern for children by 25, 20, 23 and 3.2% respectively.
Bekele, 2015 [18]	Lack of knowledge what to do (24.2%), fear of parents (21.2%), fear of the public reaction (shame) (24.2%), fear of the perpetuator (21.2) and perceived legal body is not helpful (4.5%).
Sendo, 2015 [17]	Legal body not helpful (40.7%), afraid of parent 25.9%, afraid of humiliation 14.8%, threatened by rapist 11.2% and other 7.4%
Takele, 2014 [34]	Did not know what to do 7 (28), afraid of families 12 (48), afraid of community 5 (20) afraid of perpetrator 6 (24), think legal bodies do not function 2 (8), and others 1 (4)
Bekele, 2014 [36]	Did not know what to do(33.8%), afraid the public reaction or shame (23.4%), afraid of parents (18.2%), fear revenge from perpetrator (15.6% and thought that legal body is not helpful (9.1%).
Yohannes, 2017 [52]	Feeling of shame/guilty 39 (54.9%), afraid of families reaction 28 (39.4%), didn’t know what to do 26 (36.6%), afraid of the public reaction 14 (19.4%), afraid of the perpetrator 11 (15.4%) and other 7 (9.8%)
Asfaw, 2010 [39]	68.3% did not know what to do, 41.7% afraid of parents, 36.7% ashamed of it, 30% afraid of perpetrator, 10% legal body may not helpful
Abdurashid, 2013 [40]	51(14.7%) of violence victims feel ashamed, afraid of consequence 16(4.6%), afraid of perpetrator 16 (4.6%), afraid of public reaction 19 (5.5%), and other reason 13 (3.75) to this study
Benti, 2015 [43]	Fifty one (44.7%) reported that they afraid of their parents, 49 (42.9%) afraid of public reaction, 32 (28.1%) afraid of the perpetuator, 17 (14.9%) did not know that legal body is useful in such issues
EDHS, 2016 [14]	Residence rural women (19%) than urban women (36%), setting Addis Ababa (41%), followed by women in SNNPR and Tigray (24% each) compared to Benishangul-Gumuz (9%),Women employed for cash (29%) than women who are not employed (19%), never married women (34%), those belonging to the highest wealth quintile (33%), and those who have secondary or more than secondary education (30–34%).

## Discussion

In this systematic review and meta-analysis, more than one third of the study participants were not disclosed their experience of violence. This is consistent with other similar systematic review and meta-analysis [54, 56, 57]. This high pooled prevalence of non-disclosure implies: (i) the need of strengthening women empowerment for their right, (ii) the influence of cultural belief and fear associated with domestic violence related disclosure, (iii) though the government works on women and girls’ empowerment, this study indicate the needs of evaluating its effectiveness. This may be attributed to: first lack of service provider at the grass root level. Studies showed the association of service availability and help seeking needs of women and girls [10, 19–23, 33, 39, 40]. This lack of quality services, in turn to, other psycho-social impacts including to the extent of death [31]. In the absence of effective rehabilitative and psycho-social support, women and girls survivors of violence have been reluctant to report and get the help they seek against the perpetrators [30, 31]. Second, the culture norm of the society such as: perception of violence as normal or not serious, shame and embarrassment and fear associated with domestic violence disclosure affect the life of women and girls in their need of help seeking. Culture and social norms are rules or expectations of behavior within the society to maintain individuals’ preference to follow [54–56]. The socialization process, which determines gender role, is partly responsible for the subjugation of women in Ethiopia. In the process of upbringing, boys are expected to learn and become self-reliant, major bread winners, and responsible for different activities, while girls are brought up to conform, be obedient and dependent, and specialize in indoor activities like cooking, washing clothes, fetching water and caring for children [2, 15, 26–29, 31]. Third, however, the government work with different partners for the prevention and control of violence, the findings of this systematic review and meta-analysis indicate the needs of more work to meet the needs of survivors of violence.

As compared to similar study, the results of our finding is consistent with finding from 24 different countries which indicated that the global pooled prevalence of reporting DV to any source was 39.86% (95% CI: 39.35, 40.37) [58]. The results our finding is also supported with the World Health Organization (WHO) multi-country study reported prevalence of disclosure (34–95%). However, varies rate of disclosure was observed among different countries with prevalence ranged from 23% in Cambodia [12], 31.99% (95% CI: 30.91, 33.07) to 47.64% (95% CI: 45.80, 49.48) in India, East and Central Asia, Eastern Europe [58] and 79% in Namibia [1, 2, 12], which implies the cultural, economical variation against DV.

Regarding barrier to disclosure, fear associated with disclosure such as consequence, shame and embarrassment affected the victims’ help seeking need. Victims are often unsure of what happens if they disclose their experience of violence. In Ethiopia, women’s ability and tolerance against domestic violence is considered as her indication of strength in the process of keeping good relationship in the society and maintain her partners [26, 31, 54, 55], thus if she share to third party such as: friends, family or legal bodies; this may lead her to alienation, shame, embarrassment, or blame [20, 23]. On the other hand, from those survivor of DV and disclose their violence related experience family and friends are the commonly preferred source of support [26–28]. The remaining few study also identify police as a source of support/strategies [27]. Self-defense like fought back is also reported as defensive mechanism against DV [27]. This is consistent with a recently published review from Middle East. This study revealed that fears of further violence, loss of support and relationships, cultural expectations and family reputation as reasons why women do not seek services for domestic violence [57, 58].

### Strengths and limitations of the study

To our knowledge, this is the first systematic review and meta-analysis about the pooled prevalence of DV-related disclosure in Ethiopia. Include all studies from different setting (school/ college/university and general population), without time limitation. However, limitations like use of different measurement tool (self-developed) may affects the different diminutions of help seeking behaviours. Use of reference lists and Google Scholar to include all the available studies may have some overlooked studies. The exclusions of qualitative studies and inconsistent reported studies of associated factors inability to carry out meta-analysis may also affect the in depth of identified associated factors.

## Conclusion

More than one third of women and girls were not disclosed their experience of domestic violence. Individual’s perceptions of DV as normal/not serious, shame, embarrassment fear associated with consequences disclosure, perceptions of disclosure is not help, and knowledge of where to go and what to do were identified as barrier/ factors for non-disclosure. The finding of this study suggests the need of evaluation and strengthening the collaborative work among different sectors such as: policy-makers, service providers, administrative personnel and community leaders including the engagement of men partner. This study also suggests the needs of women empowerments (capacity building) against the traditional belief, attitude, and practice.

## Supplementary information

**Additional file 1.** Quality assessment.

## Data Availability

Not applicable.

## Abbreviations

DV: Domestic violence
EDHS: Ethiopian Demographic and Health Survey
PRISMA: Preferred Reporting Items for Systematic Review and Meta-Analysis
SPSS: Statistical Package for Social Sciences
VAWGs: Violence against Women and Girls
