# Supplementary material for: Domestic violence related disclosure among women and girls in Ethiopia: a systematic review and meta-analysis
Source: Reprod Health. 2019 Dec 23;16:184. doi: 10.1186/s12978-019-0845-z (PMC6929487; doi:10.1186/s12978-019-0845-z)
Supplement: Supplementary file 1 — Additional file 1. Quality assessment. [file 12978_2019_845_MOESM1_ESM.docx]

Additional file 1 Quality assessment

| Author, year | Quality domain | | | | | | | Overall Score |
| --- | --- | --- | --- | --- | --- | --- | --- | --- |
|  | **Selection**  **(Max score=5)** | | | | **Comparability**  **(Max=2)** | **Outcome**  **(Max=3)** | |  |
|  | 1) Representativeness of the sample:  a) Truly representative of the average in the target population* (all subjects or random sampling)  b) Somewhat representative of the average in the target population* (non-random sampling)  c) Selected group of users.  d) No description of the sampling | 2) Sample size:  a) Justified & satisfactory*  b) Not justified. | 3) Non-respondents:  a) Comparability between respondents and non-respondents characteristics is established, and the response rate is satisfactory*  b) The response rate is unsatisfactory, or the comparability between respondents and non-respondents is unsatisfactory.  c) No description of the response rate or the characteristics of the responders and the non-responders. | 4) Ascertainment of the exposure (risk factor/barrier):  a) Validated measurement tool. **  b) Non-validated measurement tool, but the tool is available or described.*  c) No description of the measurement tool. | 1) The subjects in different outcome groups are comparable, based on the study design or analysis. Confounding factors are controlled.  a) The study controls for the most important factor (select one)*/barriers  b) The study control for any additional factor*  c) no control | 1) Assessment of outcome  a) Independent blind assessment **  b) Record linkage**  c) Self report *  d) No description. | 2) Statistical test:  a) is clearly described, appropriate, & measurement of association is presented, including confidence intervals & probability level (p value)*  b) is not appropriate |  |
| WHO, 2005 | b (+1) | a(+1) | a (+1) | b(+1) | a (+1) | c (+1) | a(+1) | 7 |
| Gossaye,2003 | b (+1) | a(+1) | a (+1) | b(+1) | a (+1) | c (+1) | a(+1) | 7 |
| Shanko,2013 | b (+1) | a(+1) | a (+1) | b(+1) | a (+1) | c (+1) | a(+1) | 7 |
| H/mariam,2008 | b (+1) | b(+0) | b (+0) | b(+1) | a (+1) | c (+1) | a(+1) | 5 |
| Misganaw,2013 | b (+1) | b(+0) | a (+1) | b(+1) | a (+1) | c (+1) | a(+1) | 6 |
| Bekele,2015 | b (+1) | a(+1) | a (+1) | b(+1) | a (+1) | c (+1) | a(+1) | 7 |
| Sendo,2015 | b (+1) | b(+0) | a (+1) | b(+1) | a (+1) | c (+1) | a(+1) | 6 |
| Mihrka,2016 | b (+1) | b(+0) | a (+1) | b(+1) | c(+0) | c (+1) | a(+1) | 5 |
| Takele,2014 | b (+1) | b(+0) | a (+1) | b(+1) | a (+1) | c (+1) | a(+1) | 6 |
| Nimani,2015 | b (+1) | a(+1) | a (+1) | c(+0) | c (+0) | c (+1) | a(+1) | 4 |
| Bekele,2014 | b (+1) | a(+1) | a (+1) | b(+1) | a (+1) | c (+1) | a(+1) | 7 |
| Worku,2002 | b (+1) | b(+0) | a (+1) | b(+1) | a (+1) | c (+1) | a(+1) | 6 |
| Adinew,2017 | b (+1) | b(+0) | a (+1) | b(+1) | c (+0) | c (+1) | a(+1) | 5 |
| Assefa,2010 | b (+1) | a(+1) | a (+1) | b(+1) | a (+1) | c (+1) | a(+1) | 7 |
| Abdurashid,2013 | b (+1) | b(+0) | a (+1) | b(+1) | a (+1) | c (+1) | a(+1) | 6 |
| Tadesse,2004 | b (+1) | a(+1) | a (+1) | b(+1) | c (+0) | c (+1) | a(+1) | 6 |
| Shimekaw, 2013 | b (+1) | a(+1) | a (+1) | b(+1) | c (+0) | c (+1) | a(+1) | 6 |
| Benti,2015 | b (+1) | a(+1) | a (+1) | c(+0) | c (+0) | c (+1) | a(+1) | 5 |
| Yigzaw,2005 | b (+1) | a(+1) | b(+0) | c(+0) | c(+0) | c (+1) | a(+1) | 4 |
| Semahegn,2013 | b (+1) | a(+1) | a (+1) | c(+0) | c (+0) | c (+1) | a(+1) | 5 |
| EDHS, 2016 | b (+1) | a(+1) | a (+1) | b(+1) | a (+2) | c (+1) | a(+1) | 8 |

**Note:** a, b, c and d: indicated to which values are allotted.
